# Functional characterization of *WRKY46* in grape and its putative role in the interaction between grape and phylloxera (*Daktulosphaira vitifoliae*)

**DOI:** 10.1038/s41438-019-0185-8

**Published:** 2019-09-01

**Authors:** Feng-Pan Wang, Pan-Pan Zhao, Lei Zhang, Heng Zhai, Yuan-Peng Du

**Affiliations:** 1 State Key Laboratory of Crop Biology, Key Laboratory of Biology and Genetic Improvement of Horticultural Crops (Huang-Huai Region, Ministry of Agriculture), College of Horticulture Science and Engineering, Shandong Agricultural University, Tai-an, 271000 Shandong China; 2 Key Laboratory of Optoelectronic Devices and Systems of Ministry of Education and Guangdong Province, College of Optoelectronic Engineering, Shenzhen University, Shen-zhen, 518060 Guangdong China; 3 Guangdong Provincial Key Laboratory for Plant Epigenetics, College of Life Sciences and Oceanography, Shenzhen University, Shen-zhen, 518060 Guangdong China; 4 College of Biological and Enology Engineering, Taishan University, Tai-an, 271000 Shandong China

**Keywords:** Metabolism, Transcriptional regulatory elements

## Abstract

WRKY transcription factors are involved in defense responses caused by biotic stresses. Phylloxera (*Daktulosphaira vitifoliae* Fitch), a pest widespread in viticulture, elicits transcriptional reprogramming of plant defense-associated components, such as regulons related to WRKYs and salicylic acid (SA) signaling. In this study, we characterized WRKY46, a WRKY transcription factor responsible for phylloxera attack, and revealed the molecular mechanism for WRKY-mediated defense responses to phylloxera. qRT-PCR and GUS staining analyses revealed that WRKY46 is induced in response to phylloxera damage and mechanical wounding. VvWRKY46 is a nuclear-localized transcription factor that activates its downstream target *VvCHIB* by direct protein–DNA interaction. Regulons involved in the SA-mediated defense response were regulated during incompatible interactions between “1103 Paulsen” rootstock and phylloxera. In addition, *WRKY46* exhibited a higher transcript abundance in “1103 Paulsen” than in “Crimson Seedless”, regardless of whether the plants were infected with phylloxera. Furthermore, the enhanced expression of *VvWRKY46* significantly attenuated phylloxera attack and delayed nymph development of composite grape plants. In summary, we demonstrated that WRKY46 plays a role in the SA-mediated defense-regulatory network by directly binding to the downstream structural gene *VvCHIB*. The phylloxera-responsive gene *WRKY46* was identified, which could improve the understanding of the basic mechanism of grapevine in response to phylloxera.

## Introduction

Plants often face multiple biological threats in highly variable environments, so co-survival strategies offer constitutive and induced resistance against microbial pathogens and herbivores. Two well-documented immune systems, pathogen-associated molecular pattern (PAMP)-triggered immunity (PTI) and effector-triggered immunity (ETI), have been reported to play important roles in plant defense responses^1^. PTI and ETI are innate immunities that synergistically activate plant defense responses to prevent the invasion of organisms^2,3^. In complex immune systems, several plant hormones, especially salicylic acid (SA), jasmonic acid (JA), and ethylene (ET), act as signaling molecules that trigger downstream defense responses^4–6^. Interestingly, the SA signaling pathway is often involved in biotroph-induced defense responses, while JA and ET are usually responsible for the immune response associated with necrotrophic pathogens and herbivores^7^. As a mobile signaling hormone, SA is able to initiate long-lasting and systemic immune responses to microbial pathogens, nematodes, aphids, and chewing-type herbivores^8,9^. An in-depth study of the genetic components can provide an improved understanding of the fine mechanisms of SA-mediated immune regulatory networks^10^. Stylet-feeding pests such as aphids, whiteflies, thrips, and parasitic nematodes can induce SA-mediated plant defense responses via mechanical damage and salivation^4,5^. Moreover, infestation of silverleaf whiteflies can cause local and systemic accumulation of SA-responsive genes in *Arabidopsis*^4^. In addition, expression profiling revealed that SA-inducible genes are activated when plants are under attack by phloem-feeding insects and nematodes^5^.

In the ETI defense response, *Resistance* (R) genes are critical for the perception and recognition of effectors delivered by the corresponding pathogens^11,12^. A previous study reported that when SA was abnormally degraded to catechol, tomato plants carrying *Mi-1* lost partial resistance to parasitic nematodes, and exogenous applications of an SA analog completely restored the resistance^13^. Furthermore, with upregulated pathogenesis-related (PR) genes, tomato pretreated with SA is highly resistant to root-knot nematode juveniles^14^. Studies conducted by Du et al.^15^ and Elhamahmy et al.^16^ confirmed the positive effects of SA applications on reducing pest population densities among crop plants. In addition to R genes, a large number of defense-related components are known to be involved in SA-mediated plant immune-regulatory networks, particularly the transcription factor WRKY. Previously, WRKY transcription factors were reported to act as multilevel regulators of plant defense responses to phytopathogenic organisms^17^. For example, overexpression of OsWRKY89 results in increased levels of SA and increased resistance to white-backed plant hoppers^18^. Similarly, a chrysanthemum WRKY transcription factor, CmWRKY48, inhibits the growth of aphids in stable overexpression plants^19^. In contrast, nematodes were found to thrive in host plants (tomato and *Arabidopsis*) when S1WRKY45 and AtWRKY23 appeared to be hijacked^20,21^. However, there is limited information on how WRKYs modulate the SA-mediated defense signaling cascade.

A report by Verk et al.^22^ provided additional evidence that AtWRKY28 and AtWRKY46 act as upstream regulators of SA metabolism, directly binding to the W-box (C/TTGAC/T) within the *ICS1* and *PBS3* promoters. In addition to SA biosynthesis, WRKY transcription factors are involved in interactions with other defense-related elements. Mitogen-activated protein kinases (MAPKs) posttranslationally phosphorylate pathogen-induced WRKY proteins to modulate SA-mediated immune responses^23^. In addition, WRKY33 is involved in the synthesis of antimicrobial camalexin by targeting the promoter of PHYTOALEXIN DEFICIENT3 (*PAD3*)^24^. The *Arabidopsis* Nonexpressor of PR gene 1 (NPR1) is essential for the correct induction of *PR*s in the NPR1-dependent defense response^25^. Yu et al.^26^ confirmed that certain WRKY genes act upstream of *NPR1*, whereas Wang et al.^27^ and Kim et al.^28^ documented that NRP1 directly affects the expression of *WRKY* genes in SA-mediated defense responses, suggesting a multilevel regulation of WRKY genes during plant defense responses. In addition, WRKYs can act as direct regulators of *PR*s independently of NRP1 or other components^29^.

Grape phylloxera is a worldwide pest that feeds only on Euvitis subgenera species. This insect feeds on the roots of susceptible grape (*Vitis* spp.) cultivars, leading to the formation of root galls called nodosities^30^. However, there is limited literature on the biological and molecular interaction between phylloxera and its grapevine host. In the phylloxera-resistant rootstock “Borner”, phylloxera attack alters the transcript abundance of genes that are related to the defense response, genes that encode hypersensitive response (HR) proteins, genes involved in the biosynthesis of phytoalexins, and genes that encode transcription factors^31,32^. In fact, preliminary GeneChip assays revealed that phylloxera infestation significantly affected the expression levels of *WRKY* genes in different grapevine genotypes^15^.

In this study, the grape WRKY gene Vv*WRKY46* was induced by phylloxera infestation on plants of the cultivar “Crimson Seedless” and of the rootstock “1103 Paulsen”. Further investigation revealed that *WRKY46* is involved in the SA-mediated immune response by targeting downstream defense-associated genes.

## Materials and methods

### Plant materials and phylloxera

The table grape cultivar “Crimson Seedless” (C133-199 x Emperor) and the rootstock “1103 Paulsen” (1103P, *Vitis berlandieri* x *Vitis rupestris*) were used in this study. “Crimson Seedless” is susceptible to phylloxera, while 1103P is mildly resistant. All plants were cultivated in vitro on 1/2-strength MS solid media with half the amount of macronutrients; the media were supplemented with 20 g/L of sucrose, 7.0 g/L of agar powder, and 0.2 mg/L of the phytohormone IBA. The plants were grown at 25 °C/20 °C under a 16 h/8 h (light/dark) photoperiod. Shoot cuttings with a minimum of one bud and leaf were used for monthly subcultures. Well-rooted plantlets were transplanted into plastic pots (10.5 cm height × 10.5 cm diameter) filled with a mixture of 50% peat and 50% perlite. The pots were subsequently covered with plastic bags for 1 week and then incubated under constant conditions of 25 °C/20 °C and a 16 h/8 h (light/dark) photoperiod.

Five-year-old self-rooted grapevine plants (Crimson Seedless) were used to determine the tissue-specific expression of *VvWRKY46*. Different tissues were collected in the 2016 growing season and immediately frozen in liquid nitrogen; at least three biological replicates were included. Whole fruit were sampled at 4 weeks after fruit set. The fruit skin and pulp were dissected from the harvested fruit.

*Arabidopsis* ecotype Columbia (Col) seedlings were transplanted into square pots filled with peat and vermiculite (1:1, v/v) in a growth chamber maintained at 20 °C/18 °C and with a 10 h/14 h (light/dark) photoperiod.

Excised tertiary roots of the cultivar “Kyoho” [“Campbell Early” (4 × ) × “Centennial”] were used to rear phylloxera (*Daktulosphaira vitifoliae*) as described by Granett et al.^33^. To collect nymphs for inoculation, the phylloxera insects were reared in Petri dishes as described previously by Granett et al.^33^. The incubator was strictly sealed using parafilm to prevent any phylloxera from escaping.

### Obtaining transgenic grape roots

Shoot cuttings of grape plantlets with a minimum of two buds and leaves were prepared for infection under aseptic conditions. *Agrobacterium rhizogenes* strain C58C1 harboring the 35S:VvWRKY46-GFP plasmid was freshly grown on resistant plates at 28 °C for 2 days (d). A 20 -ml culture of liquid broth containing kanamycin (50 mg/L) and rifampicin (20 mg/L) was inoculated with the above *A. rhizogenes* and incubated at 28 °C overnight with shaking at 150 rpm. The bacterial cells were collected and then resuspended to A_600 _= 1.0 using sterilized 1/2-strength MS liquid media containing 100 μM acetosyringone. *A. rhizogenes* infection was carried out in a 250- ml conical flask, in which the shoot cuttings were submerged in the resuspension in the dark, in which and the conical flask was shaken at 100 rpm at 25 °C for 15 min. The shoot cuttings were then blotted with sterile filter paper to remove excess *Agrobacterium* and subsequently inserted into solid rooting media (1/2-strength MS medium, 20 g/L of sucrose, 7.0 g/L of agar powder, 200 mg/L of cefotaxime, and 0.2 mg/L of IBA). Independent regenerative roots were induced around the wounds of shoot cuttings at 4–7 weeks after infection. The transgenic roots were subjected to light at 480 nm when they were ~3 cm in length, and the transgenic and nontransgenic roots were discerned according to green fluorescence.

### Phylloxera inoculation

A stereomicroscope (Olympus, Japan) and a small artist’s brush were used to collect 1st-instar or 2nd-instar phylloxera nymphs on a piece of filter paper (5 cm diameter). There were 50–60 nymphs on each filter paper. The filter paper was soaked with sterile water to prevent nymphs from escaping. The plantlets that had been previously transplanted into pots for four to 6 weeks were inoculated using the newly collected young nymphs according to the method described by Granett et al.^33^. To inoculate young grape roots with the nymphs, whole roots were removed from the pots, taking care to avoid harming them. Four to five pieces of filter paper that harbored phylloxera nymphs were used to wrap the young roots, and the paper was affixed by winding them with a thin thread. The inoculated roots were put into pots and covered with moistened vermiculite. The roots of each inoculated plantlet were examined using a stereomicroscope to confirm the formation of the feeding site. According to the time course assay indicated, young phylloxera-injured roots were sampled, and the samples were frozen in liquid nitrogen and stored at −80 °C. Accordingly, the young roots of control plants were treated using a fine needle mimicking the puncture of the nymph stylet with the help of a stereomicroscope. The mechanically wounded young roots were wrapped with filter paper that was not exposed to nymphs and put into the pots, after which they were covered with moistened vermiculite. The control plants were sampled by collecting young wounded roots in accordance with the time course. The treatments corresponding to each time point included three independent biological replicates.

To investigate the mRNA level of *WRKY46* in grape roots after nodosity formation, several inoculated plants were incubated under normal conditions of 25 °C/20 °C with a 16 h/8 h (light/dark) photoperiod. One month later, the nodosities and healthy roots were collected and kept in liquid nitrogen. Roots harvested from noninoculated plants were used as controls. Each sample had at least three biological replicates.

To evaluate the effects of SA and Me-JA on phylloxera, the tertiary grape roots that were sampled from 5-year-old “Crimson Seedless” were divided into 5 -cm pieces. Both sides of the root segments were wrapped in absorbent cotton to avoid desiccation. The root pieces were then put into Petri dishes (9 cm in diameter) in an incubator at 25 °C. Before inoculation with phylloxera nymphs, SA (0.1 mM, Sigma, USA) and Me-JA (1 mM, Sigma, USA) solutions were used to moisten the absorbent cotton. Sterile water was used as the moistening agent for the mock control treatment. Each root was inoculated with 100 nymphs, which were placed on the root surface. The number of feeding nymphs was then measured 10 days post inoculation.

To evaluate the susceptibility of transgenic roots produced from “Crimson Seedless” shoot cuttings, ~50 phylloxera eggs (5 days post oviposition) were prepared to inoculate each composite plant as described by Kellow et al.^34^. Before inoculation, transgenic roots were distinguished by tying thin threads after fluorescence detection. The inoculated composite plants were put into glass bottles and incubated under constant conditions of 25 °C in the dark. One week later, the numbers of phylloxera nymphs that infested successfully were calculated per root using a stereomicroscope (Olympus, Japan).

### The total RNA extraction, qRT-PCR, and semiquantitative RT-PCR

Plant RNA was extracted via a previously described modified method^35^. The total RNA was quantified with a microspectrophotometer ND2000C (Thermo, USA) set to different wavelengths. RNA integrity was confirmed using 1% agarose gel electrophoresis. Approximately 1 μg of the total RNA was then used for first-stand cDNA synthesis using a PrimeScript^TM^ RT Reagent Kit with gDNA Eraser following the manufacturer’s protocol (TaKaRa, Japan). For qRT-PCR analysis, a stock solution of the first-stand cDNA was diluted 20–40 times with sterilized ddH_2_O. qRT-PCR was then conducted in a 96-well plate using Real Time PCR instrument (Life Technologies, USA). Each well contained a 20 μL of reaction volume consisting of 1 μL of diluted cDNA, 10 μL of Ultra SYBR Mixture (CWBIO, China), 0.5 μL + 0.5 μL of gene-specific primers (10 mM), and 8 μL of sterilized ddH_2_O. The PCR amplification procedure was as follows: initial denaturation at 95 °C for 10 min, followed by 40 cycles of 95 °C for 10 s and 60 °C for 30 s. The specificity of the PCR products was confirmed by dissociation melting curve analysis. The housekeeping genes *Vvactin* and *AtGAPDH* were used as internal references for grape and *Arabidopsis*, respectively. The values of transcript abundance were calculated using the 2^-△△Ct^ method.

For semiquantitative RT-PCR analysis, gene-specific primers were employed for PCR amplification in a 20 μL reaction volume consisting of 1 μL of cDNA stock solution, 10 μL of 2x EasyTaq PCR Super Mix, 0.5 μL each of the forward and reverse primers, and 8 μL of ddH_2_O. The reaction tubes were then subjected to the following cycling procedure: preincubation at 94 °C for 10 min, followed by 25–35 cycles at 94 °C for 30 s, 58 °C for 30 s, and 72 °C for 30 s. The housekeeping gene *Vvactin* was used as an internal control. Finally, all PCR products were separated on 1% agarose gels and detected via a DNA imaging analysis system. Three independent biological assays were conducted to ensure identical results. The gene-specific primer pairs were synthesized by GENEWIZ (Suzhou city, China) and are listed in the Supplemental Files.

### Plastid construction and Arabidopsis transformation

Standard molecular biology techniques and the incision enzyme system (TaKaRa, Japan) were used for gene cloning and plasmid construction. Grape genomic DNA and first-stand cDNA were used as templates for PCR amplification of the promoter and full-length coding sequences, respectively. The open-reading frame (ORF) of *VvWRKY46* was inserted upstream of the green fluorescent protein (GFP) driven by the cauliflower mosaic virus 35S promoter (CaMV35S). The promoter sequences of *VvWRKY46* and *VvCHIB* were subcloned into the upstream region of the reporter gene β-glucuronidase (GUS). In addition, the reporter vector p1300-GN was modified by introducing a fragment of the 3ʹ-terminus of the 35S promoter into the upstream region of the *GUS* gene, and the vector was termed mini35S:GUS. A double-stranded DNA fragment consisting of two tandem W-box motifs that were derived from the *PBS3* promoter was synthesized and inserted into the upstream region of mini35S. In the mutant reporter construct mWbox-mini35S:GUS, the core sequence TGAC of two tandem W-box motifs was replaced with AGAC, and then the double-stranded DNA fragment with mutated W-box motifs was inserted into the upstream region of mini35S. All primers used for plasmid construction are listed in the Supplemental files. Before transforming the mutant reporter construct into plant materials, the above vectors were individually transformed into *Agrobacterium tumefaciens* strain LBA4404.

To obtain stable transgenic *Arabidopsis* lines, the constructs 35S:VvWRKY46-GFP and p*VvWRKY46:GUS* were transformed into Col-0 *Arabidopsis* via the *A*. *tumefaciens*-mediated floral dip method. The resultant T0 seedlings were screened on resistant media that contained the corresponding antibiotic and confirmed via qRT-PCR. T3 homozygous transgenic lines were used for experiments.

### Subcellular localization

The overexpression vector 35S:VvWRKY46-GFP was used to carry out subcellular localization experiments. This in-fusion vector was transformed into *A*. *tumefaciens* strain GV3101 harboring the p19 helper plasmid. Square (1 cm) onion epidermal cells were cut from fresh onion bulbs and immediately submerged into a suspension of *A*. *tumefaciens* at OD600 = 0.8. After incubating for 15–20 min at 28 °C, the onion epidermal cells were removed from the suspension, and the excess *Agrobacterium* solution was blotted using dry filter papers. Infected epidermal cells were coincubated on MS media (containing 20 g/L sucrose, 100 μΜ acetosyringone, and 0.7% agar powder) for 3 days in the dark at 22 °C. A 35S:GFP blank vector was used as a positive control. GFP was detected by laser confocal microscopy at the proper excitation wavelength.

### Yeast one-hybrid assays

A yeast one-hybrid assay was performed to determine the binding activity of VvWRKY46 to the W-box motif and its putative target genes in yeast strain Y187. Double-stranded DNA fragments that consisted of two W-box motifs or their mutants were synthesized and introduced into the upstream region of the mini-promoter of HIS3, which were termed W-box-pHIS2 and mWbox-pHIS2, respectively. Grape genomic DNA was used to amplify the promoter sequences of *VvCHIB*, *VvCHIB1*, and *VvG1* in construction of the reporter vectors. The full-length coding sequence of VvWRKY46 was cloned from cDNA and inserted into the effector construct pGADT7. The effector construct pGADT7-VvWRKY46 together with the reporter vector were co-transformed into yeast cells according to the protocol provided by Clontech (TaKaRa, Japan). A pHIS2 empty vector served as a negative bait. The transformed yeast cells were screened on the selective DO medium plates [tryptophan (Trp) and leucine (Leu)-deficient, SD-T/-L]. Positive transformants were also verified by PCR amplification using gene-specific primers. Finally, the binding activity of VvWRKY46 to the W-box motifs or its target genes was tested on a selective DO medium plate [Trp, Leu, and histidine (His)-deficient, SD-T/-L/-H] supplemented with 3-aminotriazole (3-at; Solarbio) at 20 mM.

### Transient expression assays

Unexpanded leaves of grapevine (*V. vinifera* L. var. Cabernet Sauvignon) were collected during the growing season for transient expression assays. All effector and reporter constructs were transferred into *A. tumefaciens* strain GV3101 harboring a p19 helper vector. The *Agrobacterium* strain harboring individual constructs was incubated to an optical density of OD600 = 2–3, harvested by centrifugation at 5000 rpm for 5 min, and then washed with sterile water. Each strain was resuspended in inoculation buffer (10 mM MES, 2% sucrose, 0.2 mM acetosyringone, and 100 mM MgCl_2_), and mixed as indicated to the same concentration (OD600 = 1.0). The grape leaves were submerged in the suspension under negative pressure conditions (0.085 MPa) in a vacuum. The vacuum infiltration was applied for 8 min, followed by recovery for 5 min; this cycle was repeated three times until the leaves sank at one bar of pressure. After removing excess suspension with filter papers, the grape leaves were placed on moistened cotton in Petri dishes to avoid dehydration. The leaves were inoculated at 25 °C in the dark for 12 h before being transferred to normal conditions. All the leaves were subjected to histochemical staining 3 days later.

### Histochemical GUS analysis

The sampled grapevine leaves were fully immersed in staining buffer in a 100 -mL conical flask, and then the flask was sealed and stored for 12 h in the dark at 37 °C. The staining buffer consisted of 0.5 mg/mL X-Gluc (5-bromo-4-chloro-3-indolyl-β-d-glucuronide), 100 mM phosphate buffer (pH = 7.0), 0.01% Triton X-100, 10 mM EDTANa_2_, 0.5 mM K_4_Fe(CN)_6_, and 0.5 mM K_3_Fe(CN)_6_. After incubation, the reaction buffer was replaced with 75% (v/v) ethyl alcohol to decolorize the chlorophyll. Finally, the fully decolorized leaves were imaged to record the β-D-glucuronidase activity.

### Electrophoretic mobility shift assays (EMSAs)

To obtain recombinant proteins, the full-length coding sequence of VvWRKY46 was amplified via PCR from cDNA and introduced into a pET32a vector at the BamHI and SalI restriction sites. The *Escherichia coli* BL21 (DE3) strain that harbored the above construct was incubated in LB liquid media to a concentration of OD600 = 0.5. After the addition of 1 mM isopropyl thio-β-D-galactoside (IPTG), the recombinant protein of interest was induced at 16 °C or 37 °C for 6 h. The crude protein extracts were then purified using a His-tagged Protein Purification Kit according to the protocol provided by the manufacturer (His-tag, CWBIO, China), and were later used for EMSAs. Three single-stranded DNA fragments that included putative W-boxes and their complementary chains were synthesized by GENEWIZ (Suzhou city, China). Each single-stranded DNA was then labeled with biotin using an EMSA Probe Biotin Labeling Kit (Beyotime, China). The reverse complementary single-stranded oligonucleotides were equally mixed and annealed using a thermal cycler (Thermo, USA) to form double-stranded DNA according to the temperature-gradient descent strategy. Wild-type and mutated DNA probes without biotin labeling served as competitors. All the primers used in this assay are listed in Table S1.

The binding reaction mixtures contained 1 mg of purified recombinant protein and 1 μL of 10 × gel shift binding buffer (50% glycerol, 50 mM MgCl_2_, 5 mM EDTA, 10 mM DTT, 500 mM KCl, 250 mM HEPES, pH 7.4) in a total volume of 10 μL. One microliter (0.05 μM) of biotin-labeled probe was incubated with the above components for 20 min at 24 °C. For the competition assay, the HIS-tagged protein together with the binding reaction was preincubated with an unlabeled or mutated probe for 20 min at 24 °C and then mixed with labeled probes for another 20 min. After incubation, each sample was resolved on 6% native acrylamide gels in 0.5 × TBE buffer (44.5 mM Tris-base, 44.5 mM boric acid, 1 mM EDTA) for 1–2 h at 100 V. The gel was transferred to a nylon membrane (Positively CHGD Nylon Transfer Membrane, GE Healthcare, UK) for chemiluminescent detection using a LightShift^TM^ chemiluminescent EMSA Kit (Thermo, USA) according to the manufacturer’s protocol.

## Results

### Expression of *WRKY46* is rapidly induced by phylloxera attack and wounding

**Fig. 1:**
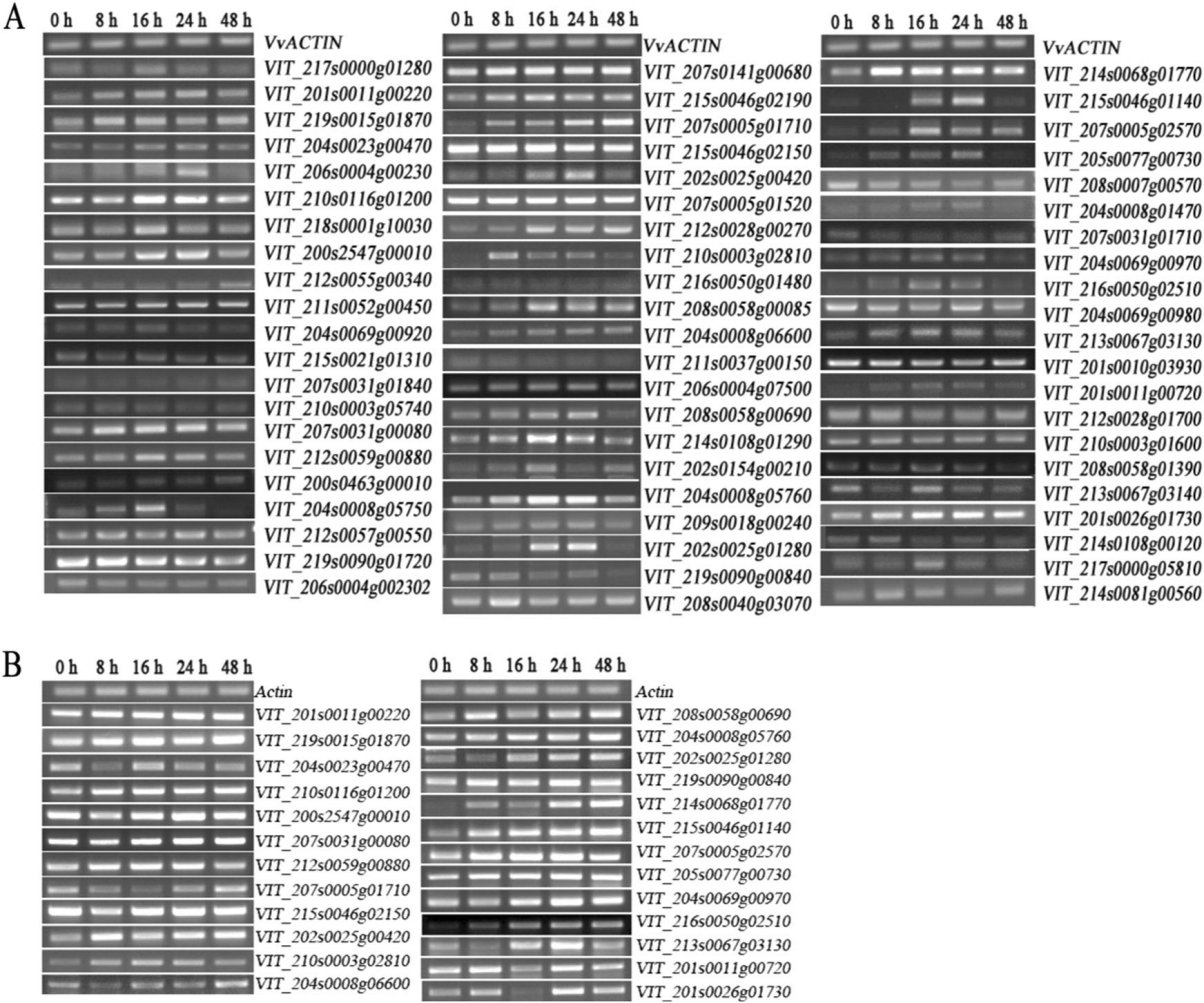
The transcript abundance of grape *WRKY* genes in roots of “Crimson Seedless” grape and rootstock 1103P caused by phylloxera attack according to semiquantitative RT-PCR. *VvActin* is used as an internal standard

The genome-wide expression profile of the grape WRKY transcription factor genes in response to phylloxera infestation was investigated for the phylloxera-susceptible cultivar “Crimson Seedless” as well as the phylloxera-resistant rootstock 1103P. The changes in the transcript abundance of the grape WRKY transcription factor genes of both genotypes revealed their putative involvement in the interactions between grape and phylloxera (Fig. 1a, b). The transcript levels of *VvWRKY*33 (VIT_208s0058g00690), *41* (VIT_202s0025g01280), *45* (VIT_214s0068g01770), *46* (VIT_215s0046g01140), and *72* (VIT_201s0026g01730) in “Crimson Seedless” increased and peaked at 24 h, with an abrupt decrease in the subsequent 48 h after inoculation (Fig. S1). However, the mRNA levels of above WRKY genes continuously increased at 48 h after inoculation (Fig. S2). Therefore, it is confirmed that the grape *WRKY* genes are widely involved in the interaction between phylloxera and its host species (Fig. 1a, b).

**Fig. 2:**
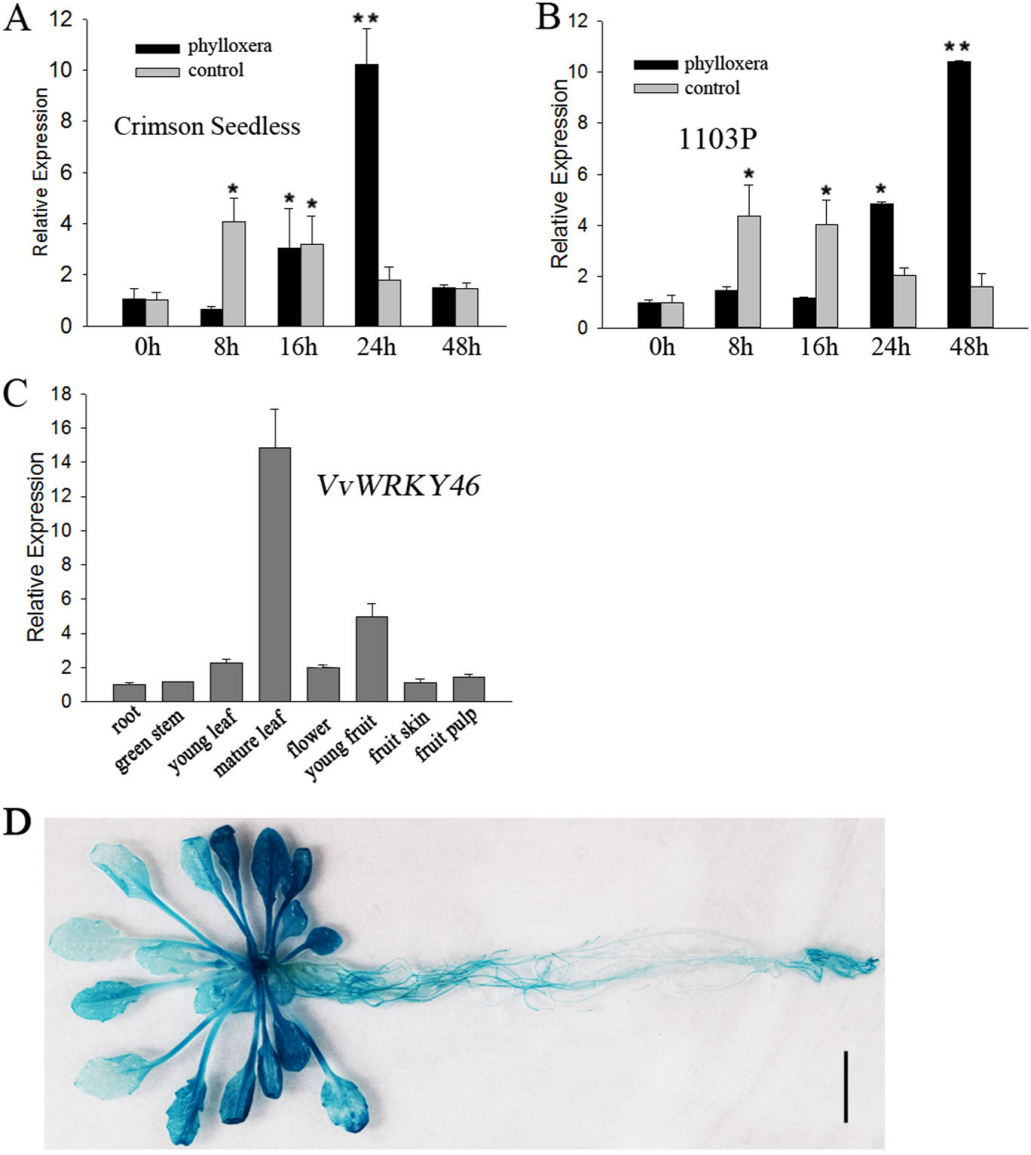
Expression pattern of *WRKY46* in response to phylloxera attack and wounding. **a**, **b** The time course of transcription levels of *WRKY46* in “Crimson Seedless” (**a**) and 1103 P (**b**) grape roots after being inoculated with phylloxera nymphs. The gene expression was normalized to the 0 h expression level, which was assigned a value of 1. **c** Tissue-specific expression patterns of *VvWRKY46* in “Crimson Seedless”. The data are shown as the averages of three biological replicates + SDs. **d** GUS gene initiated by the endogenous promoter of *VvWRKY46* expressed in transgenic *Arabidopsis* under normal conditions. Bar = 1 cm

To explore how and at what level *VvWRKY*s are associated with the innate immunity of grape phylloxera feeding damage, we conducted an in-depth study on the phylloxera-induced *WRKY* gene, *VvWRKY46*, to determine its role in the defense response. First, a time course assay was performed to determine the transcription level of *WRKY46* in the roots in the early stages of phylloxera attack. *VvWRKY46* was significantly upregulated in response to stimulated mock stylet puncture and phylloxera infestation in “Crimson Seedless” (Fig. 2a). Second, phylloxera attack resulted in a stronger and delayed increase in *VvWRKY46* mRNA levels compared with mechanical wounding. Therefore, we speculate that both mechanical and chemical stimuli in turn promote the activation of *VvWRKY46* expression, and that stylet secretions may play a leading role in the phylloxera–grape interaction. In addition, we also studied the expression pattern of *WRKY46* in partially incompatible interactions between phylloxera and “1103 Paulsen” (1103P) rootstock^36^. Similar results were obtained as excepted (Fig. 2b), which demonstrates the conserved role of *WRKY46* in response to phylloxera attack in different host plants. However, phylloxera-induced upregulation of *WRKY46* reached its maximum at 48 h post inoculation (hpi) in 1103P, suggesting a distinct defense response to phylloxera in resistant host plants.

To determine whether *WRKY46* is specifically expressed in grape leaves and roots naturally infested by phylloxera, transcript levels in different tissues of the grapevine cultivar “Crimson Seedless” were screened. As shown in Fig. 2c, *VvWRKY46* is ubiquitously expressed in all-studied organs, and has the highest level in mature leaves. To further determine the potential role of *VvWRKY46* in vegetative organs, transgenic *Arabidopsis* plants expressing the GUS reporter gene driven by the *VvWRKY46* endogenous promoter were obtained. *VvWRKY46* was constitutively expressed in different organs of *Arabidopsis* according to tissue-specific expression profiles in grape (Fig. 2d; Fig. S3A–F). The accumulation of GUS protein indicated that *VvWRKY46* may be highly correlated with senescent leaves (Fig. 2d), root hairs, root tips, the pericycle (Fig. S3A, F), lateral root initiation (Fig. S3A), leaf trichomes (Fig. S3E), and abscission zones (Fig. S3B, D). Interestingly, mechanical injury significantly elicited *VvWRKY46* expression around the wound (Fig. S4A–C), which is consistent with the upregulation of the transcript levels.

### Subcellular localization and transcriptional activity of VvWRKY46

**Fig. 3:**
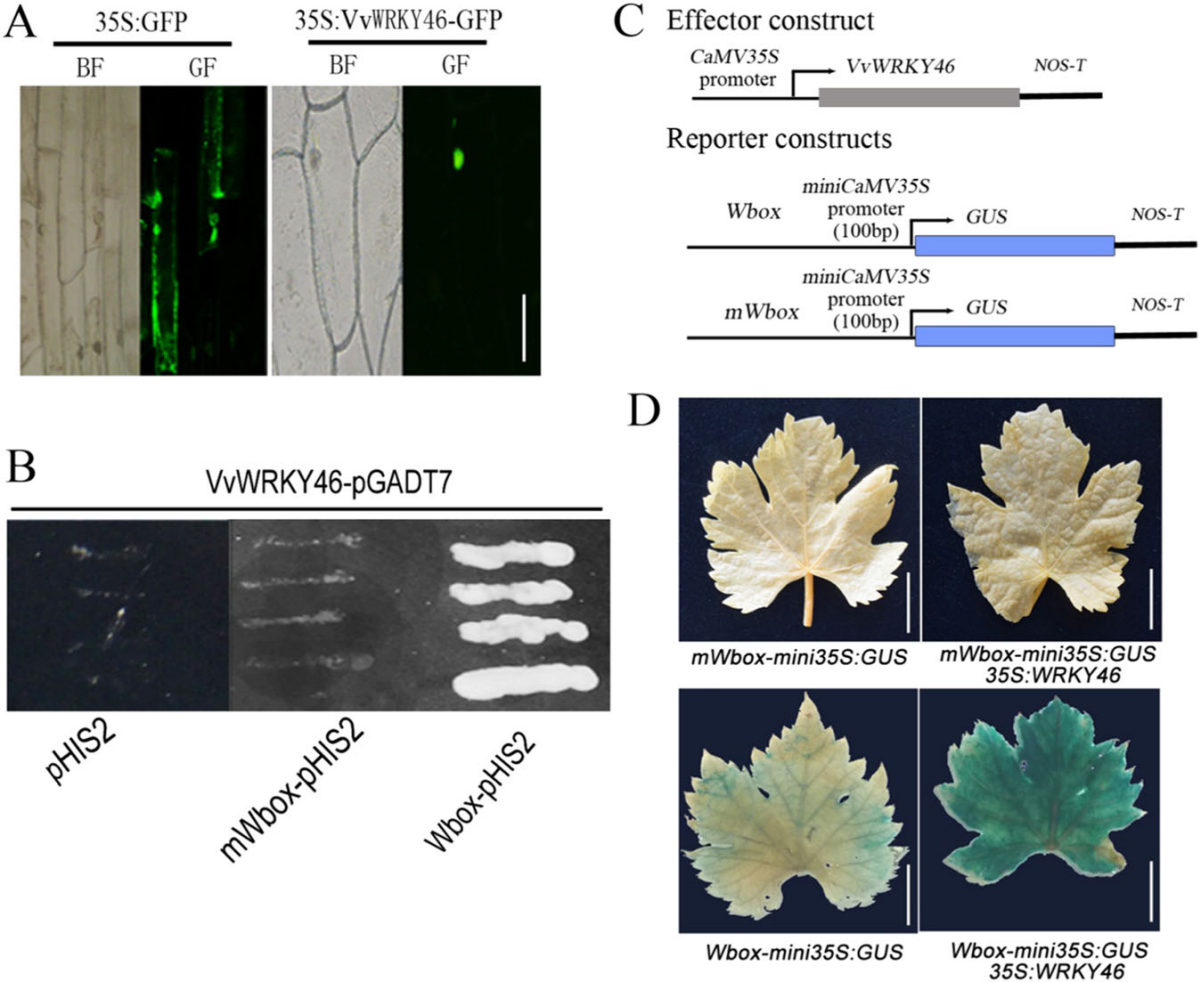
Subcellular localization and transcriptional activity of VvWRKY46. **a** VvWRKY46 was specifically localized in the nucleus of onion epidermal cells. **b** The growth phenotype of the cotransformant that harbored pGADT7-VvWRKY46 and bait vectors on a selective DO medium plate (SD-T/-L/-H) containing 20 mM 3-aminotriazole (3-at). **c** Schematic diagrams of the reporter and effector constructs used for transient expression of grape leaves via *A. tumefaciens*. W-box and mWbox motifs were inserted upstream of the mini35S promoter to initiate the *GUS* gene. **d** The GUS gene was differentially expressed in grape leaves transformed with the indicated vectors. The staining level represents the abundance of GUS protein in the leaves. Bars = 100 μm in (A), 1 cm in (**d**)

To determine the subcellular location of VvWRKY46, the full-length coding sequence was amplified via PCR and then fused to GFP driven by the cauliflower mosaic virus (CaMV) 35S promoter. The fusion constructs were then transformed into onion epidermal cells via *A. tumefaciens*. As shown in Fig. 3a, VvWRKY46-GFP fusion proteins were exclusively located in the nucleus, while the empty vector 35S:GFP was transiently expressed throughout the nucleus and cytoplasm. WRKY transcription factors are characterized by their common binding activity to the promoter sequence TTGACC/T, which is named the W-box cis-element^37^. AtWRKY46 has previously been reported to act as a positive regulator of *PBS3*, which regulates SA biosynthesis in *Arabidopsis*^22^, and the promoter sequence of *PBS3* contains W-box motifs (A/TTGACT). Thus, a tandem DNA fragment consisting of two W-box motifs, ATGACT and TTGACT, was synthesized and used to determine the transcriptional activity of VvWRKY46. In the yeast one-hybrid (Y1H) assay, cotransformants carrying VvWRKY46-pGADT7 and the W-box-pHIS2 vectors grew on SD/-Trp-His-Leu plates (Fig. 3b). However, when the W-box sequences were mutated to AaGACT or TaGACT, the yeast cells could not grow, which is similar to the results of the blank vector (Fig. 3b). Furthermore, a homologous expression assay involving *A. tumefaciens* was performed using the constructs shown in Fig. 3c. Finally, expression of the GUS gene driven by tandem W-box motifs was significantly elicited by VvWRKY46 in grape leaves (Fig. 3d), suggesting that VvWRKY46 may transcriptionally activate downstream target genes.

### Phylloxera attack triggers SA-mediated defense responses in grape roots

**Fig. 4:**
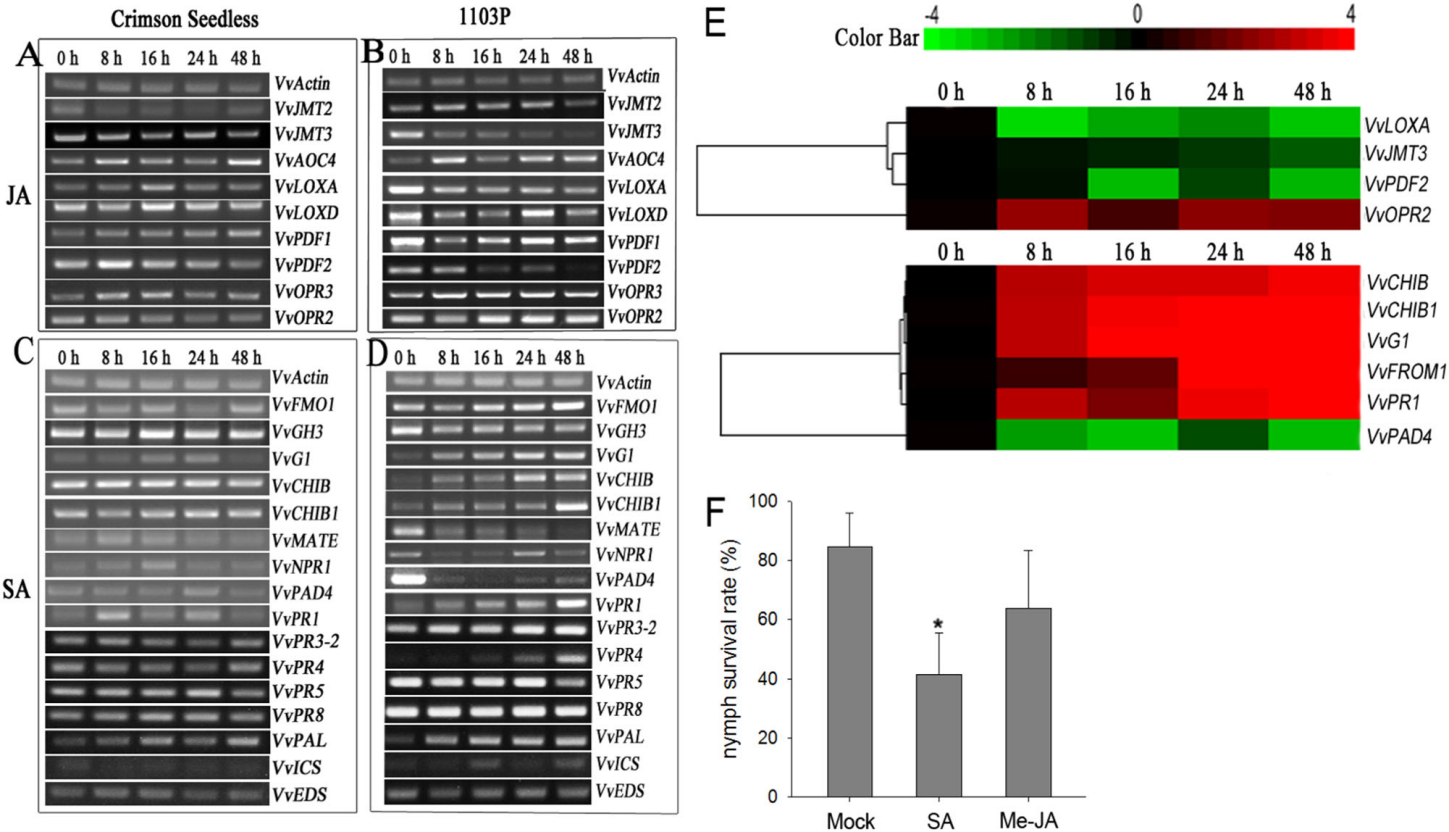
SA-mediated defense genes were inducible by phylloxera attack on grape plants. Expression of genes implicated in the SA- and JA-related signaling regulatory network in response to phylloxera attack in “Crimson Seedless” (**a**, **c**) and 1103 P (**b** and **d**) according to semiquantitative RT-PCR. *VvActin* was used as an internal control. **e** Relative expression of grape defense-related genes inducible by phylloxera attack according to qRT-PCR. The relative expression levels were subjected to hierarchical cluster analysis with the software Cluster 3.0 and TreeView 1.1.6. The color bar represents relative transcript abundances, with red representing an increase and green representing a decrease. **f** The repression effect of exogenous SA and Me-JA on nymph survival during feeding site establishment on grape woody roots. The error bars represent the SDs of three biological replicates. The asterisks in **f** indicate statistically significant differences compared with mock control: *P < 0.05

Innate immune responses occur locally and systemically in host grape plants in response to phylloxera feeding damage^15,38^. To assess the involvement of SA and JA signaling pathways in the phylloxera–grape interaction, a variety of related genes were screened for their transcript levels. The semiquantitative RT-PCR results indicated that JA-related molecular components did not show significant changes at the mRNA level in “Crimson Seedless” roots exposed to phylloxera infection (Fig. 4a). Interestingly, phylloxera attack also transcriptionally reprogrammed JA-related defense genes in partially incompatible interactions (Fig. 4b), and the expression of most of the detected genes were downregulated, except that of *VvAOC4*, *VvOPR2*, and *VvOPR3*, which are involved in JA synthesis. In contrast, several SA-related defensive genes, such as *VvNPR1*, *VvG1*, and *VvPR1*, experienced transient and slight upregulation in the time course analysis (Fig. 4c). Phylloxera infection caused a strong defense response involving the SA-related signaling pathway in the resistant host plant 1103P (Fig. 4d). Moreover, a large number of *PR* genes (*VvG1*, *VvCHIB*, *VvCHIB1*, *VvPR1*, *VvPR4*, and *VvPR3.2*) were significantly induced, implying activation of the defense response. Other SA-related genes showed unexpected expression patterns, particularly *VvGH3* and *VvNPR1*, which were downregulated. We hypothesized that WRKY46 may be involved in SA-inducible and NPR1-independent defense responses. To confirm the above results, qRT-PCR assays were performed, and similar results were obtained (Fig. 4e).

To verify whether SA and JA conferred improved resistance against phylloxera attack to the host plants, exogenous SA and Me-JA were used to pretreat grape roots in vitro before inoculation with nymphs. The nymph survival rate was then investigated to evaluate their repression effects. We found that exogenous applications of both SA and Me-JA inhibited phylloxera parasitism on the host plant (Fig. 4f). More than half of the phylloxera nymphs failed to establish feeding sites on grape roots that had been pretreated with SA compared with the mock control.

To further confirm that *VvWRKY46* is involved in the SA-mediated defense response, the overexpression 35S:VvWRKY46 construct was transformed into wild-type *Arabidopsis*. Indeed, the expression of the SA-related defense genes was upregulated in the VvWRKY46-overexpressing lines (Fig. S5), indicating that phylloxera attack-inducible *VvWRKY46* was involved in the SA-mediated defense response in plants.

### *VvHCHIB* is transcriptionally activated by VvWRKY46

**Fig. 5:**
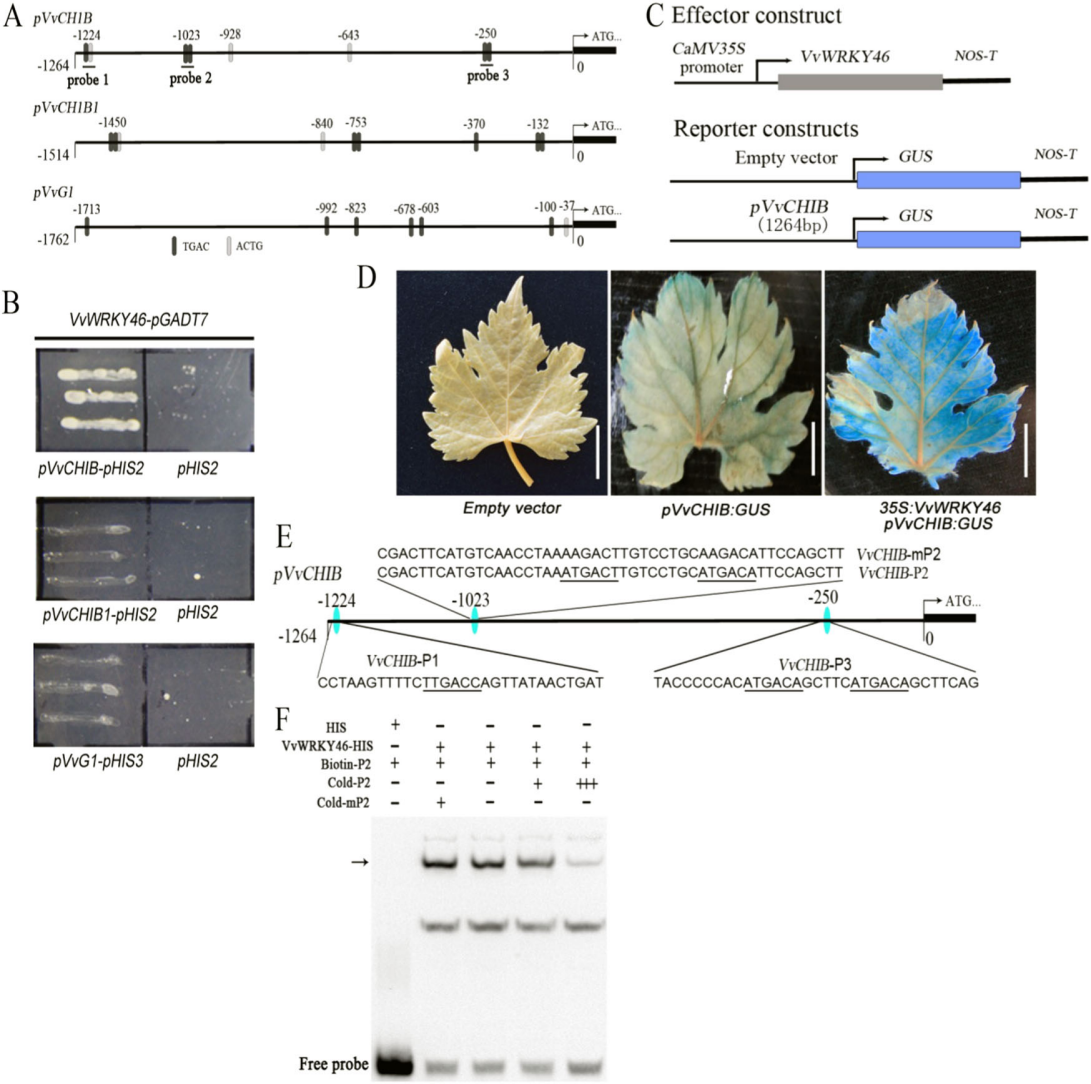
VvWRKY46 binds directly to the *VvCHIB* promoter region. **a** Promoter structure diagrams of *VvCHIB*, *VvCHIB1*, and *VvG1*. The black boxes show W-box motifs with the core sequence 5′-TGAC-3′, while the light-gray boxes show the complementary sequence of the W-box motifs (5′-ACTG-3′). **b** VvWRKY46 transcriptionally activated the promoter of *VvCHIB* in a Y1H assay. The yeast strain Y187 that harbored pGADT7-VvWRKY46 and *pVvCHIB*-pHIS2 grew well on a selective plate (SD-T/-L/-H, 20 mM 3-at). **c** Schematic diagrams of the reporter and effector constructs used for the transient expression assay. **d** Histochemical staining of GUS proteins in grapevine leaves. VvWRKY46 transcriptionally activated GUS gene expression driven by the promoter of *VvCHIB* in grapevine leaves. Each bar was 1 cm. **e** Three DNA fragments, termed VvCHIB-P1, VvCHIB-P2, and VvCHIB-P3, were used for the EMSAs. All putative W-box motifs are highlighted with black underlines. The core sequence 5′-TGAC-3′ of the W-box motif in VvCHIB-P2 was mutated into 5′-AGAC-3′, and was termed VvCHIB-mP2. **f** The binding activities of the recombinant protein VvWRKY46-HIS to wild-type and mutated probes according to an EMSA. The DNA–protein complex is indicated with a black arrow. Twenty-fold (+) and 100-fold (+++) unlabeled VvCHIB-P2 probe was added for competition assays, as well as 20-fold (+) of unlabeled VvCHIB-mP2 probe

As shown by the above results, VvWRKY46 may act as an upstream regulator of SA-induced *PR* genes in response to phylloxera attack. To determine whether VvWRKY46 regulates phylloxera-inducible *PR* gene expression, candidate W-box *cis*-elements in the promoters of *VvCHIB*, *VvCHIB1*, and *VvG1* were analyzed using the PlantCARE promoter analysis program^39^. Each of the promoter fragments of the detected *PR* genes contained a putative W-box motif (Fig. 5a); afterward, the indicated promoter sequences were cloned and subjected to Y1H assays. The reporter construct driven by the endogenous promoter of *VvCHIB* was significantly activated in yeast cells when co-transformed with VvWRKY46-pGADT7 compared with the blank vector pHIS2 (Fig. 5b). However, VvWRKY46 may exhibit weak binding activity to the promoters of *VvCHIB1* and *VvG1* (Fig. 4b). Furthermore, the homologous coexpression assay revealed that VvWRKY46 transcriptionally activated the GUS gene driven by the *VvCHIB* promoter in grape leaves (Fig. 5c, d). To test which putative W-box motif was bound by VvWRKY46, three DNA fragments (VvCHIB-P1, VvCHIB-P2, and VvCHIB-P3) were synthesized and labeled with biotin (Fig. 5a, e). Electrophoretic mobility shift assays (EMSAs) showed that the recombinant protein VvWRKY46-HIS specifically formed a protein–DNA complex with the probe VvCHIB-P2, but did not form a complex with the other two probes in vitro (Fig. 5f; S6A, B). In addition, the protein–DNA complex can be competitively inhibited via unlabeled wild-type and mutated probes, again confirming its binding activity.

### Overexpression of *VvWRKY46* alleviates the attack of phylloxera on grape roots

**Fig. 6:**
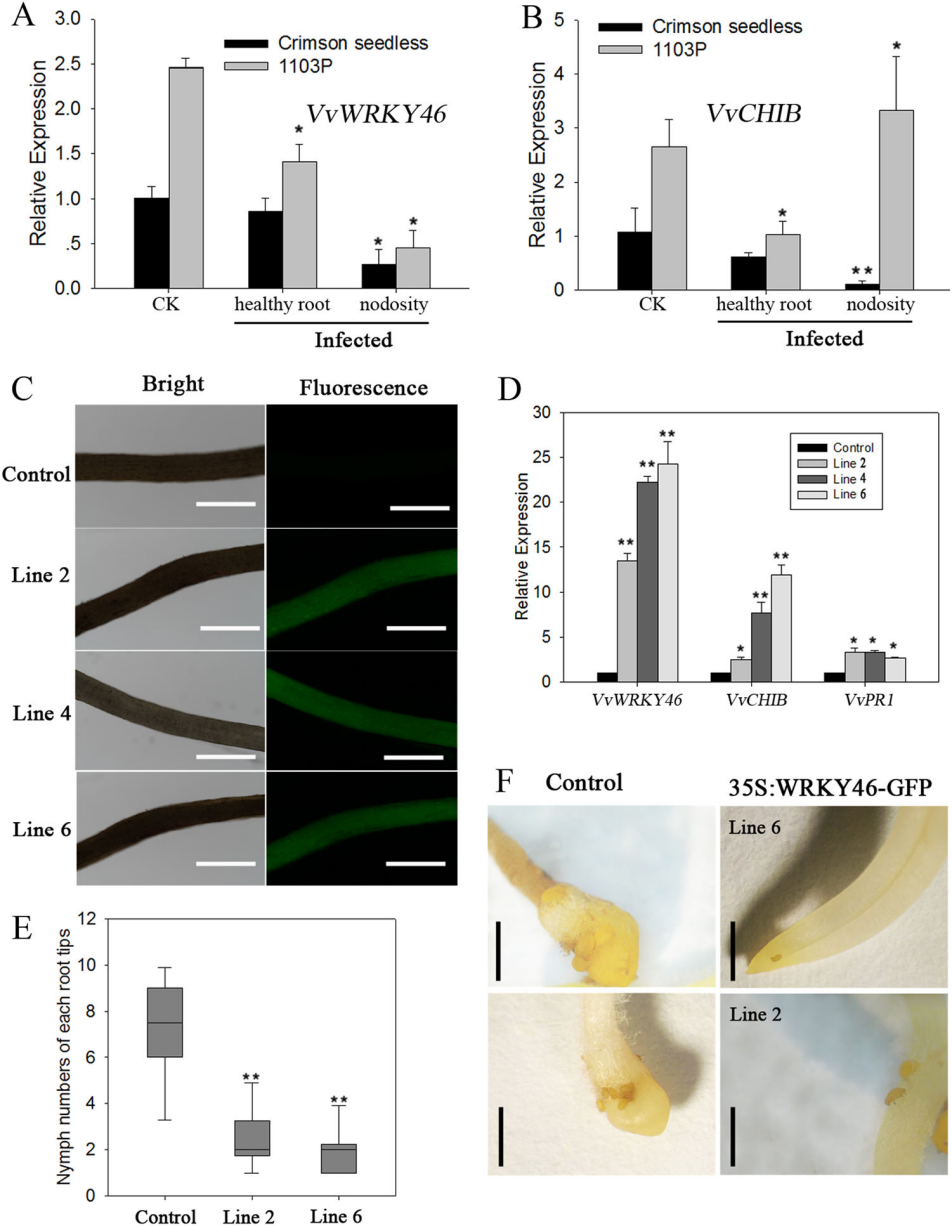
Overexpression of *VvWRKY46* alleviates the attack of phylloxera on grape roots. The mRNA levels of *VvWRKY46* (**a**) and *VvCHIB* (**b**) in infected and noninfected grape roots of “Crimson Seedless” and 1103P after nodosity formation. The gene expression levels were normalized to the noninfected expression levels, which were assigned a value of 1. **c** Transgenic roots developing on wild-type shoots of “Crimson Seedless” after infection with *Agrobacterium rhizogenes* and the expression of green fluorescence protein marker in transgenic roots at 480 nm light. The bars are 50 μm. **d** Transcript abundance of *VvWRKY46*, *VvCHIB*, and *VvPR1* in transgenic roots sampled from separate composite plants. All gene expression levels were normalized to those of the control plant (35S:GFP), which were assigned a value of 1. **e** The numbers of phylloxera nymphs that successfully fed on grape roots. The black line in each box indicates the mean value, *n* > 10. The asterisks indicate significant differences compared with the control plants according to Student’s *t* -test, **P < 0.01. **f** Micrograph of grape root nodosities induced by phylloxera nymphs at 2 weeks post inoculation. Scale bars = 100 μm

After 14 days of phylloxera infestation, the roots of the phylloxera-susceptible cultivar “Crimson Seedless” and the phylloxera-resistant rootstock 1103P formed nodosities. This finding indicates that the phylloxera successfully infested and developed on the roots. VvWRKY46 and its target gene *VvCHIB* are involved in phylloxera-induced nodosity generation in the susceptible cultivar “Crimson Seedless” (Fig. 6a, b). With respect to the mildly resistant rootstock 1103P, the mRNA level of *WRKY46* decreased at the feeding site, while *CHIB* showed higher levels of transcription than did the control plants (Fig. 6a, b); it is likely that CHIB may be associated with more WRKY46-mediated defense regulatory pathways. SA is a key molecular inducer of plant systemic acquired resistance (SAR) against a wide range of plant pathogens, including herbivores. The expression patterns of *WRKY46* and its target gene *CHIB* were detected in the healthy roots of infected plants. Interestingly, both *WRKY46* and *CHIB* responded systemically to phylloxera attack, and their expression was downregulated compared with that in control plants (Fig. 6a, b). In addition, the transcript levels of *WRKY46* and *CHIB* in 1103P were higher than those in “Crimson Seedless” regardless of whether the plants were attacked by phylloxera.

Transgenic roots were obtained by *A. rhizogenes-*mediated shoot cutting infection. Fluorescence tests on regenerated roots around the wounds were performed to check which roots were transgenic (Fig. 6c). Real-time RT-PCR confirmed that *VvWRKY46* was upregulated in the roots of composite plants and in the marker genes of its downstream target *VvCHIB* and the SA signaling component *VvPR1* (Fig. 6d). To determine whether overexpression of *VvWRKY46* resulted in enhanced resistance to phylloxera attack, phylloxera eggs (5 days post oviposition) were inoculated on the roots of line 2 and line 6 composite plants (in which *VvWRKY46* was upregulated to different degrees). The numbers of successfully infested nymphs on the transgenic roots were significantly reduced compared with those on the control plants (Fig. 6e). In addition, overexpression of VvWRKY46 caused a retardation of nymph development (Fig. 6f), and the rate of phylloxera development was affected by the expression of *VvWRKY46* in different composite plants; the development was slowest on the 6-line composite plants, in which VvWRKY46 was strongly upregulated. Thus, the positive effects of VvWRKY46 in grapevine in defense against phylloxera are strongly confirmed.

## Discussion

Previously, the results of GeneChip microarrays revealed that WRKY transcription factors are involved in phylloxera–grapevine interactions. Phylloxera damage on rootstock 140Ru (*V*. *berlandieri* *×* *V*. *rupestris*) significantly induced the expression of three *WRKY* genes, but not in “Crimson Seedless”^15^. In this study, VvWRKY46 was identified as a putative defense-related component elicited by phylloxera attack on grapevine roots. A good mechanism by which WRKY46 is involved in the plant defense response to species-specific phylloxera was studied in two grapevine genotypes. WRKY46 is differentially expressed in different grapevine species, exhibits significant susceptibility levels to phylloxera damage, and is associated with SA-mediated plant immune-regulatory networks. These findings suggests that WRKY46 may act as a key regulator and that its expression pattern is closely correlated with host susceptibility.

In general, defense-related transcription factors, such as WRKY and TGA, act downstream of SA-dependent systemic acquired resistance (SAR), which initiates later than the accumulation of the plant defense hormone SA induced by *R* genes^40^. As described above, WRKY or TGA reprograms the transcript levels of the *NPR1* and *PR* genes downstream of the SA signaling pathway by direct binding activity^28,41^. For the defense response in which WRKY46 is involved, WRKY46 has been shown to contribute to host plant resistance to biotic stresses based on the following observations: (1) pathogen-induced SA accumulation increases the transcript level of *WRKY46* (also known as WRKY48 in grape) in *Arabidopsis* and grapevine^42–44^, and (2) AtWRKY46 can promote SA biosynthesis by transcriptionally upregulating the structural gene *PBS3* in *Arabidopsis*^22^. It is speculated that WRKY46 positively regulates the SA signal transduction pathway induced by plant pathogens and may directly regulate SA metabolism through a feedback loop. However, it is unclear whether the pathogens attack simultaneously or sequentially to trigger SA accumulation and upregulation of *WRKY46* or other *WRKY* genes around the site of infection.

GUS staining and qRT-PCR assays confirmed that mechanical damage resulted in local accumulation of *VvWRKY46* mRNA and that the promoter of *VvWRKY46* was rapidly and locally transactivated around the wound. Wound-inducible WRKY transcription factors exhibit rapid and transient responses in a short period of time^45,46^. Young nymphs may be looking for the correct feeding site before puncture, and the wound caused by stylet piercing is small and limited. In contrast, *WRKY46* was incorrectly regulated by the sucking action of the phylloxera nymphs after inoculation for a long time. In piercing injuries, watery saliva secreted by phloem-feeding pests greatly promotes the induction of plant defense responses^47^. The different transcript profiles reveal a fundamental difference in defense-related components caused by mechanical damage and insect feeding^48^. It is believed that mechanical wounding and herbivory do not trigger the exact same signaling pathways in plants^49^, although a group of molecular components is involved in the downstream transduction of the two exogenous stimuli described above. Biochemical factors that are related to tissue repair and that immediately accumulate in plant cells may preferentially act as signaling molecules in response to mechanical wounding.

According to the canonical binding performance of WRKY transcription factors as previously described^37^, VvWRKY46 specifically bound to the W-box motif 5′-ATGACT-3′ instead of 5′-ATGACA-3′ or 5′-TTGACC-3′. The core sequence 5′-TGAC-3′ is essential for high-binding affinity as well as the 3′ flanking base T. With respect to upregulated genes in transgenic *Arabidopsis* plants, sequence analyses of their promoters revealed several potential binding sites for VvWRKY46. In particular, the W-box motif 5′-ATGACT-3′ bound by VvWRKY46 was identified within the promoters of *AtPBS3* and *AtPR3*, suggesting a putative cascade relationship between WRKY46 and its downstream targets. WRKY46 may have a conserved binding profile to target the *PBS3* and *PR3* genes in plants, although AtWRKY46 did not reveal the exact binding site of the *AtPBS3* promoter^22^.

Time course assays showed that *WRKY46* responded to different expression patterns of phylloxera attack in the susceptible “Crimson Seedless” and the resistant rootstock 1103P. Uehara et al.^50^ isolated and characterized several differentially expressed genes in compatible and incompatible interactions between tomato and parasitic nematodes. Due to their genomic background, defense-related components may play different roles in pathogenic attacks^51^. The transcript abundance of *WRKY46* showed a late but sustained increase in the 1103P rootstock compared with the susceptible “Crimson Seedless”. Furthermore, *WRKY46* has a higher background expression in uninfected 1103P roots than in “Crimson Seedless” and healthy roots and nodosities of infected plants. We hypothesized that 1103P specifically induces upstream *WRKY46* upregulation at the transcriptional level to initiate downstream functional genes. Indeed, the *VvCHIB* gene, which encodes chitinase and protects against chitin-containing pathogens^52^, appears to be a direct downstream target of *VvWRKY46* and exhibits a similar expression pattern, except with respect to 1103P nodosities. It cannot be ruled out that *VvCHIB* may be regulated transcriptionally by other defense-related networks responsible for phylloxera damage^15^. Interestingly, both VvWRKY46 and VvCHIB are associated with a systemic defense response induced by phylloxera attack. SA or other molecules may play pivotal roles in the signaling of the entire host plant.

## Conclusions

VvWRKY46 is characterized as a nuclear localization transcription factor responsible for compatible and incompatible interactions between phylloxera and grapevine host plants. Phylloxera attack triggered a defense response that was closely related to the SA-mediated immune regulatory network. *VvCHIB* was confirmed to be the downstream target of *VvWRKY46* by direct protein–DNA interaction. Overexpression of *VvWRKY46* significantly reduced the invasive nature of phylloxera attack and slowed the development of nymphs in composite grape plants. WRKY46 exhibits a higher transcript abundance in 1103P than in “Crimson Seedless”, regardless of whether the plants are infected with phylloxera, which means species-specific effects occur in terms of defense responses against obligate pests.

## Supplementary information

Supplementary Figures.

Supplementary table Primers for semi-quantitative RT-PCR.
