# Supplementary material for: Functional characterization of WRKY46 in grape and its putative role in the interaction between grape and phylloxera (Daktulosphaira vitifoliae)
Source: Hortic Res. 2019 Sep 1;6:102. doi: 10.1038/s41438-019-0185-8 (PMC6804638; doi:10.1038/s41438-019-0185-8)
Supplement: Supplementary file 1 — Supplementary Figures. [file 41438_2019_185_MOESM1_ESM.docx]

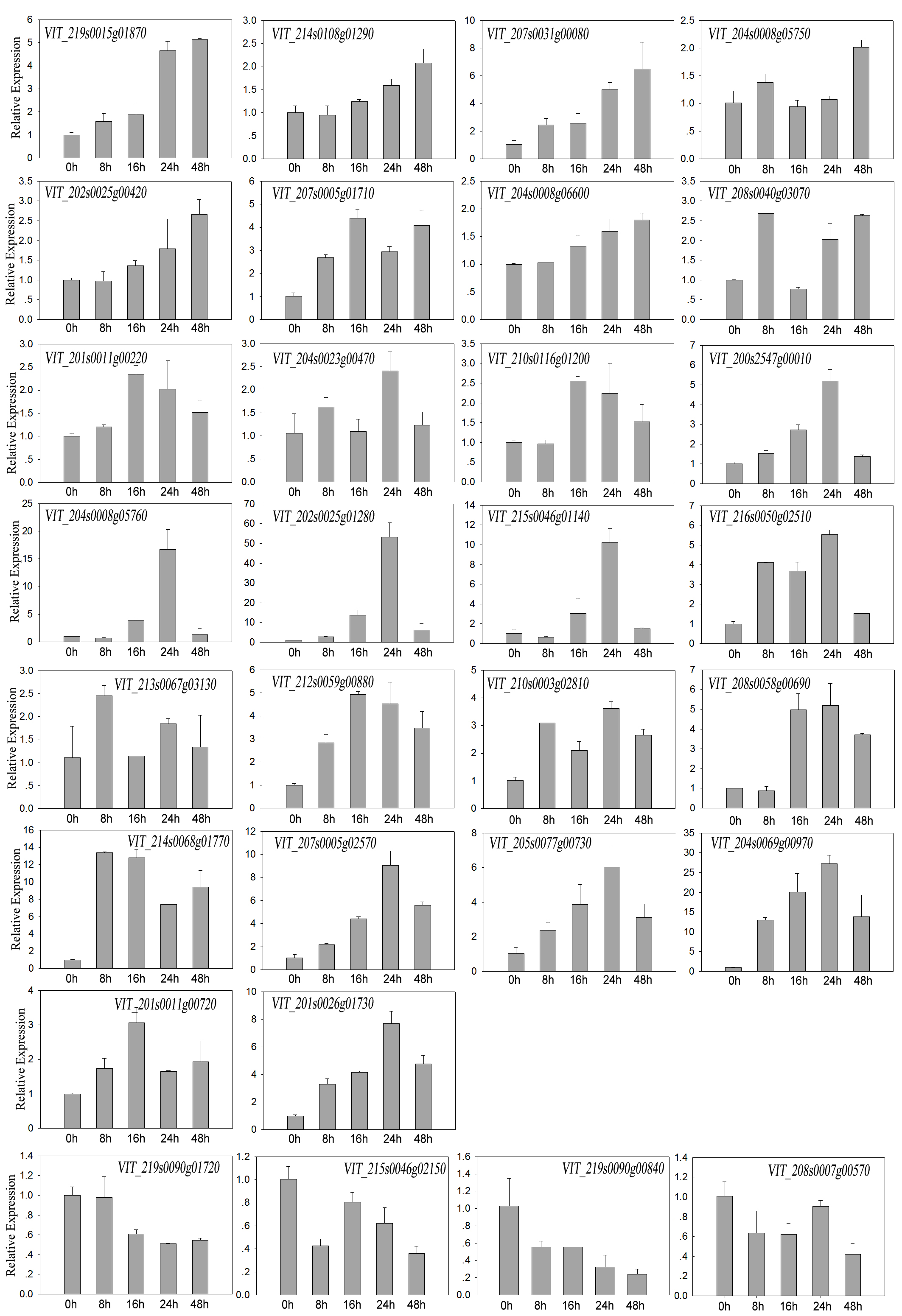


Figure S1. Relative expressions of grape *WRKY* genes in response to phylloxera attack using qRT-PCR for Crimson Seedless. *VvActin* is used as an internal control and each gene expression is normalized to the expression level at 0h.


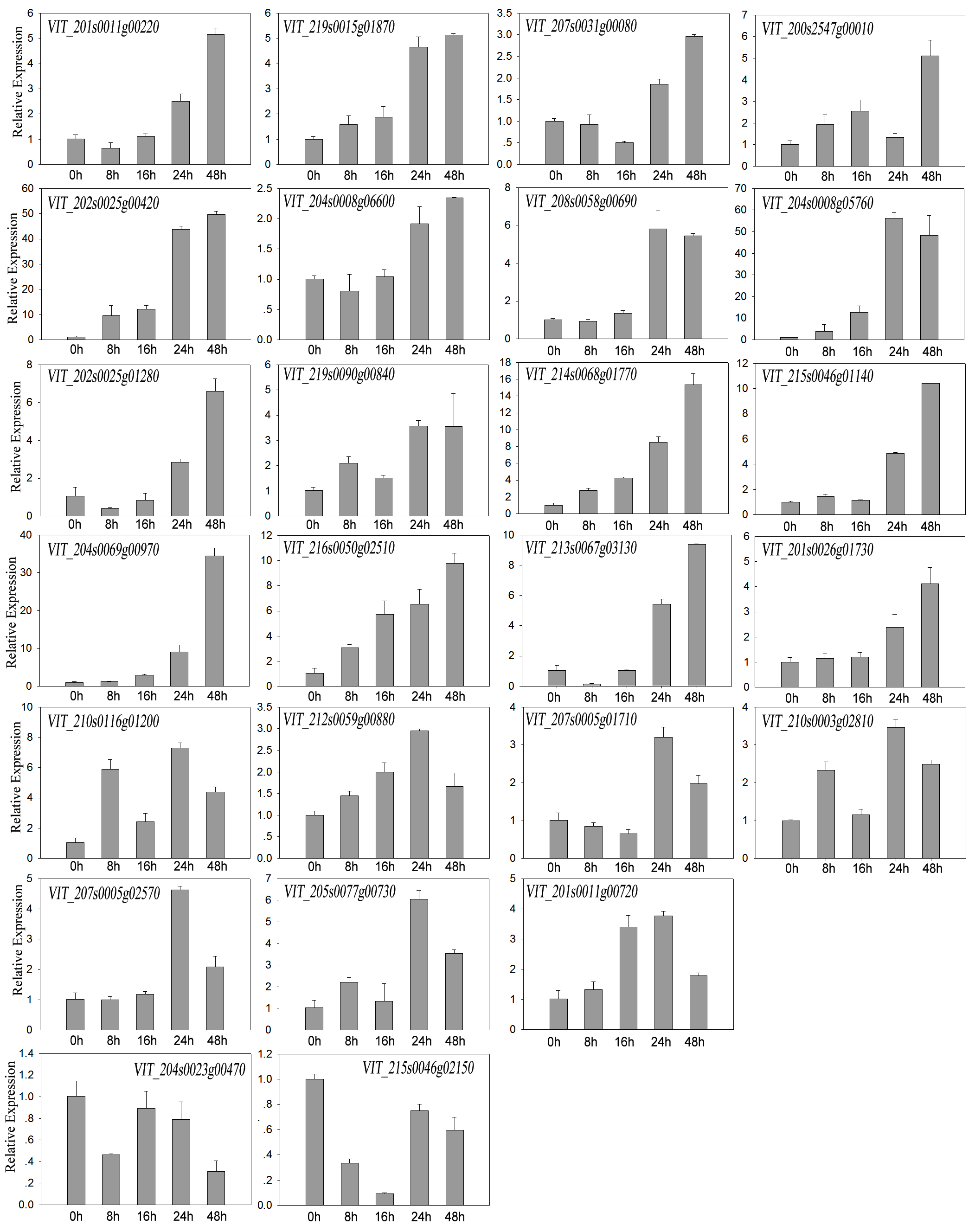


Figure S2 Relative expressions of grape *WRKY* genes in response to phylloxera attack using qRT-PCR for root stock 1103P. *VvActin* is used as an internal control and each gene expression is normalized to the expression level at 0h.


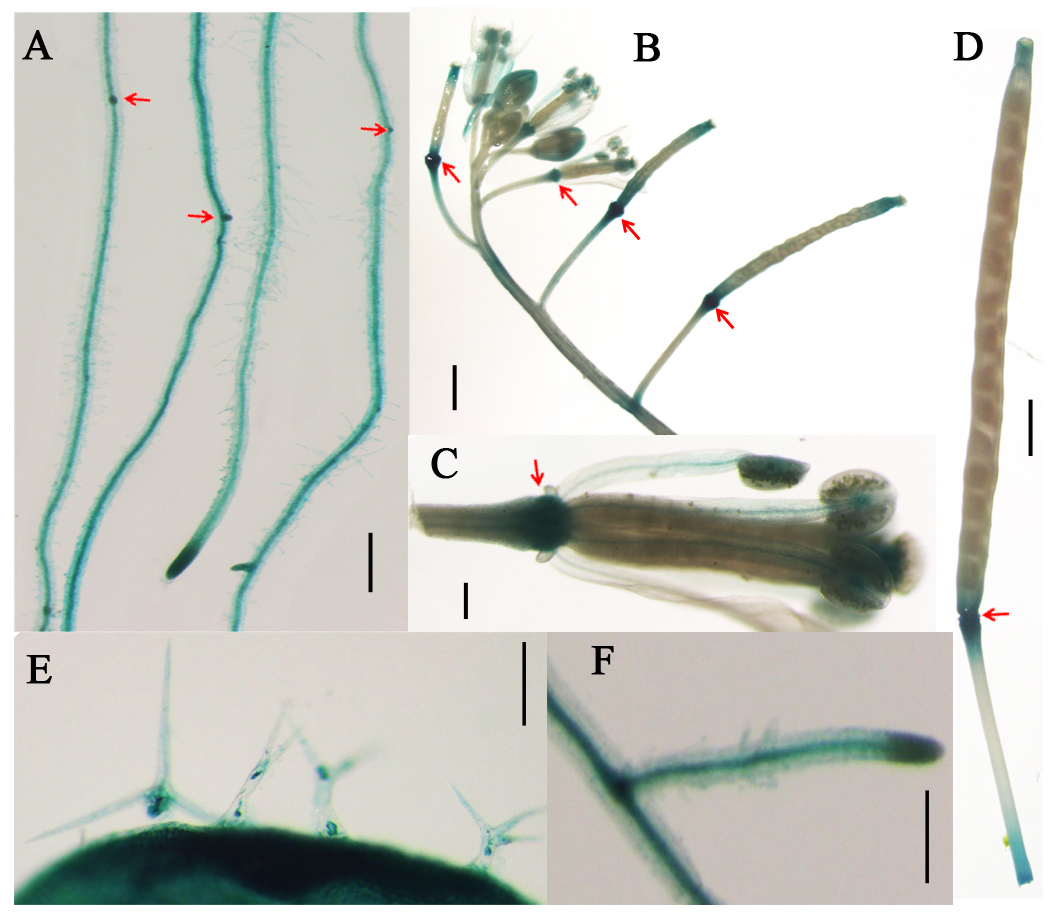


Figure S3. Accumulation of GUS protein in different organs of transgenic *Arabidopsis* plant (*pVvWRKY46*:GUS). Red arrows indicated specifically enhanced GUS expression at the site of lateral root initiation (A) and abscission zone (B-D). Bars = 0.5 mm in (A), 1 mm in (B) and (D) and 0.2 mm in (C), (E) and (F).


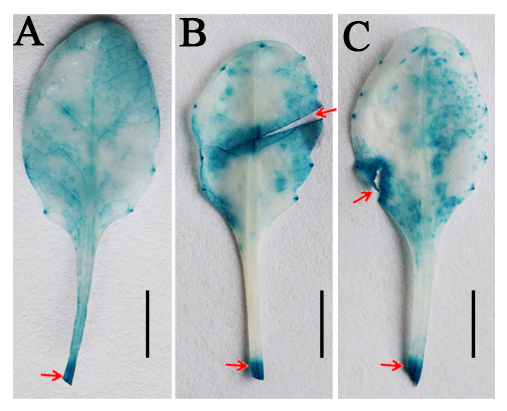


Figure S4. The promoter of *VvWRKY46* was specifically induced by mechanical wounding in transgenic *Arabidopsis* plant (*pVvWRKY46*:GUS). The healthy leaves of transgenic *Arabidopsis* plants were hurt by scratching (B) or tearing (C) as the red arrows indicated. Bars = 0.5cm.


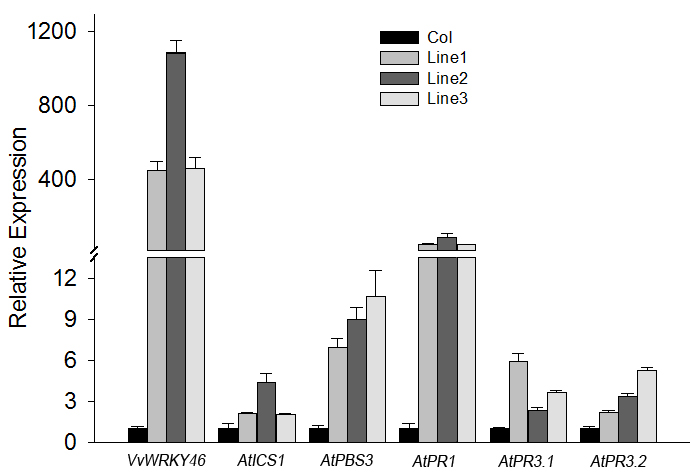


Figure S5 Transcript abundances of *VvWRKY46* and SA-related genes in transgenic *Arabidopsis* plants. Each gene expression is normalized to the Col control expression level, which is assigned a value of 1. The error bars represent the SD of three biological replicates.


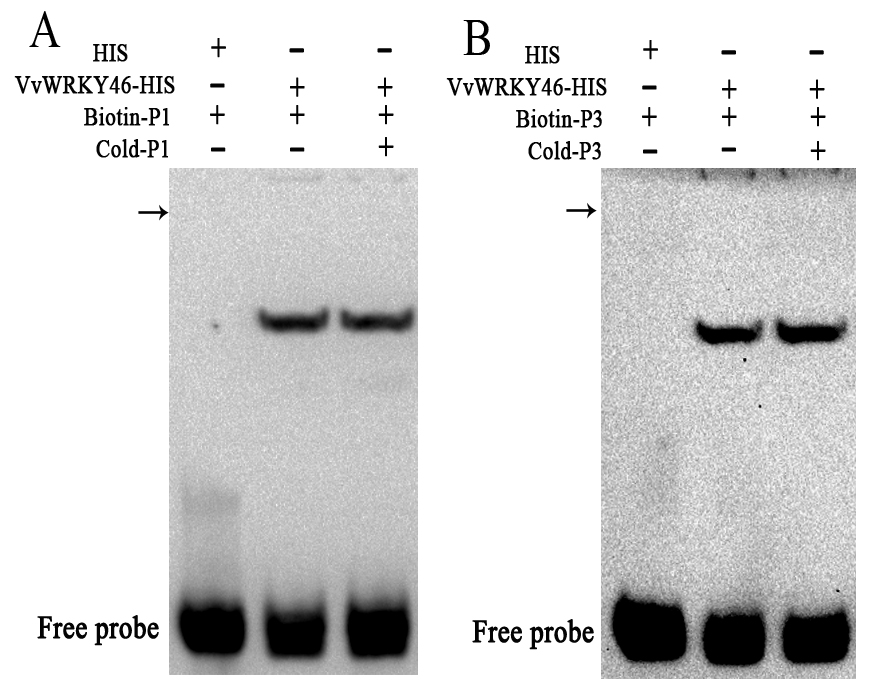


Figure S6 The binding activities of recombinant protein VvWRKY46-HIS to putative W-box elements on the promoter of *VvCHIB*. DNA-protein complex was indicated with black arrow. 20-fold (+) of unlabeled probe (VvCHIB-P1 and VvCHIB-P3) was added for competition assays.
