# Supplementary material for: Functional characterization of WRKY46 in grape and its putative role in the interaction between grape and phylloxera (Daktulosphaira vitifoliae)
Source: Hortic Res. 2019 Sep 1;6:102. doi: 10.1038/s41438-019-0185-8 (PMC6804638; doi:10.1038/s41438-019-0185-8)
Supplement: Supplementary file 2 — Supplementary table Primers for semi-quantitative RT-PCR. [file 41438_2019_185_MOESM2_ESM.docx]

Table S1 Primers for semi-quantitative RT-PCR

| Gene locus ID | Forward | Reverse |
| --- | --- | --- |
| VIT_217s0000g01280 | TCCTTGATGATGGGTAT | AGTTCTCGGTGGGCTTC |
| VIT_212s0059g00880 | GAGCCTTCGCTGTCTTC | GGTTGCTTCGGTTTGAT |
| VIT_206s0004g07500 | ATCGGATTATGGCAGTT | CTTGGGATGGTTATGAGTA |
| VIT_207s0031g00080 | ATAGCAAGCAACCCTCAT | GGAACTCTAACCACCCTCT |
| VIT_204s0069g00970 | CGAAATCAGAGCTGGACG | CAGGGATTGGATGGTTATGG |
| VIT_210s0003g01600 | GGGCTTGGAGGAAGTATGG | TTGCTGGTTTGGGTGAGG |
| VIT_219s0090g00840 | AGAGGGAGACAACCGCACAT | AGACGCCCGACCCAAATC |
| VIT_219s0090g01720 | TGATGGGTATCGGTGGCG | GTGCGGGTTTGGGTTAGA |
| VIT_208s0058g00690 | TCACAGTGCTCCATTCGC | GCCTGCTGGTGCTCGTTA |
| VIT_218s0001g10030 | CAAGAGGGTTATTTCGTT | GAGGTTGGTGGTTATGAG |
| VIT_207s0031g01840 | CAACTGGGTTTCTATTCTT | CATCTCCACCGATACTTC |
| VIT_217s0000g05810 | TCTTGATTACCACCTATGA | AGAGCGACGAGTTGATTG |
| VIT_200s0463g00010 | TCCAAGGAGCTACTACAA | CTACAGTCAGAACCCACA |
| VIT_212s0028g01700 | ACCGTAGGCTCCACATCA | GCTTCTGCCCATACTTTCT |
| VIT_210s0003g05740 | TGGAGGCGGCGGTATGTT | TAGGAGCCAAGGCGGAAG |
| VIT_202s0025g00420 | GACACTTCGTTGCCTTGC | TGGTCCTGCCCTTTCATC |
| VIT_204s0023g00470 | CAGGCACAACTGTCTCC | GCATCCCTACATTCTCAC |
| VIT_209s0018g00240 | CCAAGGGAGGAGACTATTA | GGTTCACGGATCGGTTT |
| VIT_202s0025g01280 | GGACTGTGAGCGAGGAT | CGTTATGGTGGTTCTGTTT |
| VIT_201s0010g03930 | GACTTCCAAACCCACAA | CCCATACTTCCTCCATCT |
| VIT_208s0007g00570 | GGGAAATGCCAATCAGA | GTAGGCGAAGTGGAGGT |
| VIT_215s0021g01310 | TTTCTTGGCACCATCACA | CTCCTCGCCTTCACTTTG |
| VIT_214s0081g00560 | TTTGATGGCTCTAGTTGC | TCTTCACCCTCAGTTTCC |
| VIT_204s0069g00920 | TTCACTTCCCGTTCCTC | CCCTGCTTTCCGTTAGA |
| VIT_208s0058g01390 | ATTCGCAATCTTGGATC | GCCGTGGTATGTGGTTT |
| VIT_210s0116g01200 | CGAGCCTACTACCGATGT | TGGGTGAGGTCCAATGTC |
| VIT_214s0108g00120 | TGGTAAGTCCAGCATCG | TTCATCGTTAGCGTGTC |
| VIT_212s0057g00550 | TGTGGATCGTGCTCTTGC | CTTGGATTTGTATTGCCTTT |
| VIT_215s0046g02190 | CCAAGCGAAGAAAGAATC | TCCTGTGGGTGGGCATA |
| VIT_208s0040g03070 | ACAAGCGAGCCCTCTAAG | CTGCTATCTGCCCATCCA |
| VIT_216s0050g02510 | AACCAGCAACAAGCACAAG | ATGGAACCAAACGGGAACT |
| VIT_210s0003g02810 | CTCGGAGATTTAGGTGCCA | TCCCATCTGTCCTGGTGTT |
| VIT_207s0141g00680 | GGGGAGGCTGTGGTTAGGT | TCTGCTGTTGCTGGTGGTG |
| VIT_213s0067g03140 | AGAGTGGGAACTACAAGGT | TCATAGCCAGGATAAGAGC |
| VIT_205s0077g00730 | TCATCACCAAGAGCGAAGT | GAGGTCCTCAAGAAGTCCA |
| VIT_219s0015g01870 | TCTACCACTTTGCAGGGTC | GATTCGGATTTCCTTTCAC |
| VIT_201s0026g01730 | GCAAAGATGTGCGGAGGAC | TTGGGATCTAAAGGAGACGAA |
| VIT_211s0037g00150 | ACTTCTGTTACCCAGTCTATT | CATGATTGTGCCGACTTTT |
| VIT_207s0005g01520 | CTCCCAACAGCCTAAACCA | GAAGTTGAAGCCGCTCCTC |
| VIT_204s0008g06600 | AGAGCGGACCAGAAAGACT | CTCCCTTCAACAGGACTAACA |
| VIT_201s0011g00720 | GGACGATAACGACAATCCC | TTGGTCATAAACGCAAAGC |
| VIT_214s0068g01770 | GTGGAGGCTTTGGTAGGTC | TAGGGAGCGTAGATTTGCA |
| VIT_215s0046g01140 | TCAAGGACCAAGCAGGCAAAG | TCCACTAGCACGACCGAGGC |
| VIT_213s0067g03130 | TACAACTGTCCAGCCAAGA | TATCAGCGAGATCCACCAC |
| VIT_207s0005g02570 | CCTTGCCCTCGTGCCTACT | CATGGGATTGGTGCCTTGC |
| VIT_207s0005g01710 | AGTTGGTTCGTGGTCATTT | CTCCATCTGTACCCATCTTC |
| VIT_212s0055g00340 | AAATTGATCTGTCCCTGAA | AAGACTTTGGAAATCCTCC |
| VIT_204s0008g05760 | TTTGGTAGAAGAGTTGCGTAG | CTCCATTGGTATCCGTCCT |
| VIT_204s0008g05750 | CGGTAGCCAAGGCATCACA | TCTTCTCCTGACCCGTCCC |
| VIT_207s0031g01710 | GGAAGTATGGGAAGAAAT | AAGGAACCTGGTCGTAG |
| VIT_204s0008g01470 | CATTCCTGGCATCCGAGTT | TAGAAGGGCTTTCGTGGTT |
| VIT_215s0046g02150 | TGGAGATATTGACTGGGCTAC | GCTTCTCACAGGGATGGTT |
| VIT_212s0028g00270 | CGCCTAATTCCTTGGACAC | CAGCATTGCCTCTTATCGT |
| VIT_200s2547g00010 | AAACTGCTCACCCTACTTG | ATGAATAACGGATTGTCTG |
| VIT_204s0069g00980 | GGAGATTATGGGCGGTGAT | GAGGGTGAGAAGAAGTTTG |
| VIT_206s0004g00230 | TTTTCCTGGGAGGTGGG | CTGGTTGGTGGCTGTGC |
| VIT_211s0052g00450 | AGATGGACGAGCAGATGGC | TCCGTTGAGACGACGAAAG |
| VIT_201s0011g00220 | GCCATACCTGAACACTTACC | CTCAACATGCTTACGGACA |
| VIT_208s0058g00085 | TGGGACAGGGAGGACTTCA | AACTGCTTTGGCTGGTGGC |
| VIT_216s0050g01480 | CCCATATCCACGGAACTAC | TCTTCAAACATTGGCTCAC |
| VIT_214s0108g01290 | CGTCTCGCCGACCTTCTAT | TTGGATTCTGCCACGCTCT |
| VIT_202s0154g00210 | GCAGGAAACTGAGGATAAA | CAGAACAGAGTCCCACAAA |

Table S2 Primers used in this paper

| Gene locus ID | Forward | Reverse |
| --- | --- | --- |
| RT-W46-F | TCAAGGACCAAGCAGGCAAAG | CTTTCTGTCCATATTTTCTCC |
| RT-Vvactin-F | GAGATTCCGTTGTCCAGAAGTC | CAATGTTGCCATAGAGGTCCTT |
| RT-AtICS1-F | AACAGTGTCATCTGATCGTAATC | TTAAACTCAACCTGAGGGACTG |
| RT-AtPBS3-F | GTACCGATCGTGTCATATGAAG | TCACATGCTTGGTTATAACTTGC |
| RT-AtPR1-F | AGCTCAAGATAGCCCACAAG | TAGTTGTTCTGCGTAGCTCC |
| RT-AtPR3.1-F | GGCAAAGCCTTGGACGAGAA | CTCGTGCGAACATTAGTGGT |
| RT-AtPR3.2-F | AGGTCCCATTCAACTATCT | GGAGTCTGTTCGGTCATC |
| RT-AtGAPDH-F | TGAAATCAAAAAGCTATCAAGG | CATCATCCTCGGTGTATCCAA |
| RT-VvFMO1-F | GCCTTTCCGTAGTGTAATAGA | ATCTGGTGGTCTTCTTCATAA |
| RT-VvG1-F | GAGACTCTACGACCCTAACC | CTTCATTTCCAACCGCAAC |
| RT-VvGH3-F | TGTCGGTCTTGTAGATGTG | CAGGCTTCACTATCCTAATC |
| RT-VvCHIB-F | TGTCTGGCTCAAACTTCACA | GAACACGACCTGCTGATGT |
| RT-VvCHIB1-F | GCATAGAAATGAAGGGAGTTG | TGACATTATGGCAGGAAGGC |
| RT-VvMATE-F | GGTGGCTTTCTACTCTTTCCT | TGACTTCAGCAGCATTCGGG |
| RT-VvNPR1-F | TGTTAGCGAGGTGTATTGA | GCATTTCTACCGTCTGGCG |
| RT-VvPAD4-F | CCCATCTCCAATCCACTTCTT | AGCTTTCCCAACATAATCC |
| RT-VvPR1-F | AGTCCATTAGCACTCCTTTGTT | TCTGGGCGTAGGCAGCCACAGT |
| RT-VvPR3.2-F | TTCAAGTAGCGGCAGTGGTA | ATTCTTCCCAGCACAACTCG |
| RT-VvPR4-F | TGAGAATAGTGGACCAATGC | CTGTAAGATGACCTTTGGCG |
| RT-VvPR5-F | GTCCCTGCTTCTCAGTCTCG | CTGCGCCTGGTTGTATGCCT |
| RT-VvPR8-F | AGACTAACACCCAGTTCACA | CCAAGTATGGAGGCGTGATG |
| RT-VvPAL-F | CCTGGCAACTCTATACTTC | AGATCCGATCACCTACAA |
| RT-VvICS-F | GTTTCAACGCACTCACTC | CTGGAGACGTAGAATCCC |
| RT-VvEDS-F | ATGAGCAAAGGCACCGACAG | TCCGTTCCCAAATATCCC |
| W46-6HIS-F | TCCCCCGGGTATGGAAATGGCCAGGGAGT | GACTCGAGTTGGAAGAATTCAGGATTAT |
| W46-GFP-F | GTCGACATGGAAATGGCCAGGGAGTGG | GGTACCTTGGAAGAATTCAGGATTAT |
| W46-AD-F | AGTGAATTCCACCCATGGAAATGGCCAGGGAGTGG | ATCGATGCCCACCCTTGGAAGAATTCAGGATTAT |
| pW46-GUS-F | AACTGCAGCATCATAGGTAAACCAAAGAC | GCTCTAGACCAAGGAAATGGCCTTGTAAG |
| pVvG1-F | GAATTCTGACTTCTTCTGCTGCCCAT | GAGCTCGCATTAACACCGAGATTTAG |
| pVvCHIB-F | GAATTCATAGCTTTGGAGATGTTGGT | GAGCTCGGAATTGGCGATAGAAGATC |
| pVvCHIB1-F | CCCGGGTTGTCCTCCTCTATGATGAA | GAGCTCTACACTAAGCTAGAGATTGC |
| CHI1B-P1-F | CCTAAGTTTTCTTGACCAGTTATAACTGAT | ATCAGTTATAACTGGTCAAGAAAACTTAGG |
| CHI1B-P2-F | CGACTTCATGTCAACCTAAATGACTTGTCCTGCATGACATTCCAGCTT | AAGCTGGAATGTCATGCAGGACAAGTCATTTAGGTTGACATGAAGTCG |
| CHI1B-mP2-F | CGACTTCATGTCAACCTAAAAGACTTGTCCTGCAAGACATTCCAGCTT | AAGCTGGAATGTCTTGCAGGACAAGTCTTTTAGGTTGACATGAAGTCG |
| CHI1B-P3-F | TACCCCCACATGACAGCTTCATGACAGCTTCAG | CTGAAGCTGTCATGAAGCTGTCATGTGGGGGTA |
| mini35S-F | GCTCTAGAACGCACAATCCCACTATCCT | CGGGATCCCGTGTTCTCTCCAAATGAA |
| Wbox-F-GUS | GGCTAATTTGACATTTATGGTCGTGTTTGACGTTGTAGCTTAAGAAATGACTTTTAACTCT | CTAGAGAGTTAAAAGTCATTTCTTAAGCTACAACGTCAAACACGACCATAAATGTCAAATTAGCCTGCA |
